# The Neurobiological Underpinnings of Obsessive-Compulsive Symptoms in Psychosis, Translational Issues for Treatment-Resistant Schizophrenia

**DOI:** 10.3390/biom13081220

**Published:** 2023-08-05

**Authors:** Licia Vellucci, Mariateresa Ciccarelli, Elisabetta Filomena Buonaguro, Michele Fornaro, Giordano D’Urso, Giuseppe De Simone, Felice Iasevoli, Annarita Barone, Andrea de Bartolomeis

**Affiliations:** Section of Psychiatry, Laboratory of Translational and Molecular Psychiatry and Unit of Treatment-Resistant Psychosis, Department of Neuroscience, Reproductive Sciences and Dentistry University Medical School of Naples “Federico II”, Via Pansini 5, 80131 Naples, Italy

**Keywords:** psychosis, obsessive-compulsive disorder, antipsychotics, selective serotonin reuptake inhibitors, clozapine, treatment-resistant schizophrenia, postsynaptic density, synapsis

## Abstract

Almost 25% of schizophrenia patients suffer from obsessive-compulsive symptoms (OCS) considered a transdiagnostic clinical continuum. The presence of symptoms pertaining to both schizophrenia and obsessive-compulsive disorder (OCD) may complicate pharmacological treatment and could contribute to lack or poor response to the therapy. Despite the clinical relevance, no reviews have been recently published on the possible neurobiological underpinnings of this comorbidity, which is still unclear. An integrative view exploring this topic should take into account the following aspects: (i) the implication for glutamate, dopamine, and serotonin neurotransmission as demonstrated by genetic findings; (ii) the growing neuroimaging evidence of the common brain regions and dysfunctional circuits involved in both diseases; (iii) the pharmacological modulation of dopaminergic, serotoninergic, and glutamatergic systems as current therapeutic strategies in schizophrenia OCS; (iv) the recent discovery of midbrain dopamine neurons and dopamine D1- and D2-like receptors as orchestrating hubs in repetitive and psychotic behaviors; (v) the contribution of N-methyl-D-aspartate receptor subunits to both psychosis and OCD neurobiology. Finally, we discuss the potential role of the postsynaptic density as a structural and functional hub for multiple molecular signaling both in schizophrenia and OCD pathophysiology.

## 1. Introduction

Almost 25% of schizophrenia patients suffer from obsessive-compulsive symptoms (OCS), while approximately 12% meet the diagnostic criteria for obsessive-compulsive disorder (OCD) [1,2,3,4,5,6]. Notably, diagnosis of OCD, age at onset before 20 years, male sex, and comorbidity of autism spectrum disorder have been associated with a higher risk of schizophrenia [7]. OCS severity in cognitive performance has been shown to correlate with early age at onset and increased frontoparietal and cingulate gyrification as potential markers of early neurodevelopmental deficits. The presence of OCS in schizophrenia patients could be responsible for making the overall clinical presentation even more complex than schizophrenia and may further worsen the patient’s functioning [8]. Specific patterns of OCS emergence and duration in schizophrenia may be liable for different clinical trajectories [9]. Current definitions of OCS have been criticized for their overly inclusive nature, which limits differential diagnosis and more precise prognostic stratification. The co-existence of OCS and psychotic symptoms in schizophrenia patients from the beginning could result in a greater probability to suffer from treatment resistance [10]. Nearly 30% of people with schizophrenia suffer from the severe illness known as treatment-resistant schizophrenia (TRS) [11,12,13,14,15]. Despite available clinical, neuroimaging, and neurophysiological findings, little is known about the neurobiological underpinnings of OCS presentation in schizophrenia patients. The proposed OCD and schizophrenia dysfunctional brain circuits overlap at various levels [16], and three neurotransmitter systems, dopaminergic [17], glutamatergic [18,19,20], and serotonergic, with their multiple interactions are regarded as a potential major player in the pathophysiology of both disorders.

In this scenario, some challenging hypotheses have been raised, and the present review has been proposed to address a critical appraisal of the following issues:

(1) Do schizophrenia core symptoms (positive, negative, and cognitive) share common dysfunctional brain networks with OCD relevant to dopamine–glutamate–serotonin interplay?
(2) Do schizophrenia and OCD pathophysiology share some presynaptic and postsynaptic mechanisms and do genetic findings support a potential overlapping?
(3) How does dopamine–glutamate–serotonin interaction support translational research to identify new therapeutic strategies?

We will attempt to provide a molecular framework of OCS presentation in schizophrenia patients and possibly relevant inferences for their treatment, especially for those patients not responding to available antipsychotics.

## 2. Obsessive-Compulsive Symptoms in Schizophrenia

### 2.1. Clinical Framework for Multiple Neurotransmitters’ Implication of OCS in Schizophrenia

OCS have been described at different stages during the clinical trajectory of schizophrenia [21]: (i) before the onset of psychosis, as an independent diagnosis of OCD, or as part of the at-risk mental states [22,23,24]; (ii) simultaneously with the onset of psychosis; (iii) after the onset during the chronic course of the illness or after starting second-generation antipsychotics (SGA) treatment [21,25]. Although the clinical diagnosis of OCD was not associated with later conversion to psychosis, the severity of OCS in young people at ultra-high-risk for psychosis was associated with a more acute symptomatic presentation, including more severe depression and suicidality [23]. A study evaluating the 5-year course of OCS/OCD in patients with first-episode schizophrenia found no associations with a more severe course of psychotic symptoms and relapse. In this context, comorbidity with OCD was associated with more severe depressive symptoms, social dysfunction, and worse premorbid functioning [24]. Schizophrenia patients with OCS have more severe psychotic symptoms, lower quality of life, significant pronounced deficits in specific cognitive domains, more impairment in social functioning, and higher general psychopathology compared to their counterparts without OCS [21,23,26,27,28,29,30]. A prospective cohort study conducted in a Danish population of more than three million people showed that the presence of a previous diagnosis of OCD was associated with an increased risk of developing schizophrenia and schizophrenia spectrum disorders later in life [31]. Taken together, these results suggest that OCS may not only influence the course, treatment response, and prognosis of psychotic symptoms, but that schizophrenia and OCD may share a common etiological pathway [31].

The differentiation of obsession and compulsion, the core symptomatology of OCD, from delusions and delusionally guided repetitive behaviors is decisive to define a potential subgroup of patients affected by schizophrenia and referred to as schizo-obsessive [32]. Based on the variety of clinical presentations, both “schizotypic OCD” [1,33] and “schizo-obsessive” subtypes of psychosis [34,35,36,37,38] have been proposed [34]. The challenge to differentiate clinically psychotic symptoms and OCS consolidates the idea that schizophrenia and OCD may rely, at least in a specific subpopulation of patients, on a putative common neurobiological substrate [31,39]. The concomitant presence of schizophrenia and OCS in one clinical entity is challenging for its treatment and perhaps is contributing to the onset of TRS [38]. Deeper insight into the putative mechanisms underlining the pathophysiology of OCS in schizophrenia could help disentangle the pathophysiology of this clinical manifestation and may shed light on more efficacious treatment.

### 2.2. Genetic Findings of OCD in Schizophrenia: Relevance for Serotonergic, Dopaminergic, and Glutamatergic Systems

The possible interrelationship between schizophrenia and OCD has been explored by considering, in both preclinical and clinical settings, the possibility of common genetic factors underpinning the two disorders.

Devi and co-workers reported high rates of OCS in schizophrenia patients’ first-degree relatives, suggesting possible genetic contributions and differences in the neurobiology of the disease [29]. The glutamatergic signaling may exert a relevant role in linking OCS and schizophrenia, as indicated by the association between sequence variations in the *solute transporter family* (*SLC1A1*) gene on chromosome 9p24, which encodes the *neuronal excitatory amino acid glutamate transporter 1 (EAAC1*), and susceptibility to SGA-induced OCS [40]. A significant interaction effect of the single nucleotide polymorphisms (SNP) rs7525948 of the *disc large associated protein 3 (DLGAP3)* gene—coding for a post-synaptic scaffolding protein of glutamatergic synapses, also known as SAP90/PSD95-associated protein 3 (SAPAP3)—and the rs2228622 variant of the *SLC1A1* gene was demonstrated in the SGA-induced OCS. The *DLGAP3*/*SLC1A1* interaction might be involved in the susceptibility to develop OCS in antipsychotic-treated schizophrenia patients [41] (Table 1). Pointing toward the evidence of association of the *SLC1A1* gene with SGA-induced OCS in schizophrenia patients [40], Poyurovsky and collaborators suggested the possibility of a genetic predisposition, which is unmasked by SGA treatment with a different load [38]. The authors also suggest that a genetic predisposition with a differential load might exist in schizophrenia patients who exhibit OCS, probably unrelated to atypical antipsychotic agents [38]. In a cohort of 250 patients with chronic schizophrenia under clozapine treatment, the impact of SNPs in *SLC1A1*, *Glutamate Ionotropic Receptor Kainate Type Subunit 2 (GRIK2)*, and *N-methyl-D-aspartate receptor subunit 2B (GRIN2B)* genes on OCS was assessed [38]. SNPs of *SLC1A1* (rs2228622) and *GRIN2B* (rs890) genes as well as the interaction between the two genes showed a tendency for association with OCS in schizophrenia patients receiving clozapine [38]. Preclinical studies also indicate a role of glutamatergic transmission in OCD [42,43]; mice with genetic deletion of DLGAP3, highly expressed in the striatum, showed increased anxiety and compulsive grooming behavior [42]. Electrophysiological, structural, and biochemical studies in mutant mice revealed defects in corticostriatal synapses, underlining their implication in obsessive-compulsive behavior. Preclinical evidence in transgenic mice with inactivation of the *SLIT and NTRK Like Family Member 5 (Slitrk5)* gene have been shown to develop OCD-like behavior attenuated by the administration of selective serotonin reuptake inhibitors (SSRI) fluoxetine. *Slitrk5* knock-out (KO) mice showed a reduction of 20–60% in NR2A and NR2B subunits of N-methyl-D-aspartate receptor (NMDAR) expression, leading researchers to hypothesize that it is involved in the development of OCS by regulating glutamatergic neurotransmission [43]. *Slitrk5* KO mice showed also selective hyperactivation of the orbitofrontal cortex (OFC) and decreased striatum volume associated with reduced dendritic spines, contributing to a possible corticostriatal abnormal connectivity [43]. In summary, specific genetic risk factors predispose patients with schizophrenia to develop OCS induced by antipsychotic treatment, and risk-conferring polymorphisms in genes linked to the glutamatergic system, such as *SLC1A1*, *DLGAP3*, and *GRIN2B,* have been identified [10] (Table 1). Variants of the *catechol-O-methyltransferase (COMT)* gene, which represents a key regulator of dopaminergic neurotransmission and possibly a pharmacological target, has also been considered as a genetic predisposition factor to OCS in schizophrenia [44,45]. On the other hand, considering that OCD treatment is mainly represented by SSRI, the studies on candidate genes for this condition are focused on serotonin neurotransmission. Although the results are mixed due to phenotypic heterogeneity, the polymorphisms of both *serotonin transporter-linked promoter region* (*5-HTTLPR)* and *serotonin receptor (5-HT)_2A_* (*HTR2A*) genes appear to be more consistently related to OCD [46]. The 5-HTTLPR polymorphism associated with affective psychosis [47] does not appear to be, by itself, a susceptibility factor for schizophrenia [48], with mixed results in influencing the response to SGA in patients with schizophrenia patients [48,49]. No significant association was found between the rs6313 (102T/C gene) and rs6311 (1438 A/G gene) polymorphisms of the *HTR2A* gene and schizophrenia [49]. However, the comparison of genotype and allele frequencies of polymorphisms between groups of patients with or without a family history of schizophrenia showed that the frequencies of the T and A alleles were significantly higher in the group with a family history of schizophrenia [50]. Evidence of a correlation between the SLC6A4 (gene encoding for serotonin transporter—SERT) locus and OCD [51,52] has been reported, whereas Genome-Wide Association Studies (GWAS) found no association between the *SLC6A4* gene and schizophrenia [53]. Interesting data from GWAS are related to the *Calcium Voltage-Gated Channel Subunit Alpha1 I (CACNA1I)* gene whose SNP rs5757717 seems to be commonly associated with schizophrenia and OCD, suggesting that genetic factors could cause some of the comorbidity for these two disorders [54]. Structural magnetic resonance imaging (MRI) scans suggested that *SLC6A4* might be related to deficits in structural schizophrenia brain networks [55], indicating that functional genetic research on this polymorphism must be further explored [56]. The association between genes affecting the glutamatergic, dopaminergic, and serotoninergic neurotransmission could suggest that neurotransmitters overlap within the cortico-striatal-thalamo-cortical circuit (CSTC) in both OCD and schizophrenia conditions [57].

### 2.3. Neuroimaging Studies: Points of Convergence between OCD and Schizophrenia, Potential Biological Underpinnings for OCS in Schizophrenia

Brain abnormalities as unveiled by imaging studies may involve common regions in schizophrenia and OCD: the frontal lobe, the basal ganglia, the thalamus, and the cerebellum [59]. Functional neuroimaging studies have shown reduced neural activation in the dorsolateral prefrontal cortex (DLPFC) and right caudate of schizophrenia patients with and without OCS compared to healthy controls [60,61]. Moreover, the comorbidity of schizophrenia with OCD does not change abnormal language processing, as revealed by the reduced lateralization in left prefrontal language areas (i.e., BA44/45, inferior frontal gyrus) and diminished inter-hemispheric functional connectivity. These observations support the theory that abnormalities in language processing are more representative of schizophrenia than OCD [62]. Although patients with schizophrenia compared to those with OCD showed some similarities in spontaneous brain activity in the parietal and occipital lobes, they exhibited different patterns of brain activity in the frontal, temporal, parietal, occipital, and insular regions, albeit with more significant abnormalities in functional connectivity in schizophrenia patients, implying different neurobiological mechanisms [63]. Magnetic Resonance Spectroscopy (MRS) has been used to explore the putative profile of brain metabolites in OCD and could be informative to understand differences and overlaps with schizophrenia. A recent systematic review evaluated the role of glutamate and white matter abnormalities in OCD and schizophrenia suggests a progressive dysfunction in brain regions related to working memory that could be considered a potential transdiagnostic marker [64,65,66]. A review examined 28 proton magnetic resonance spectroscopy (^1^H-MRS) studies comparing OCD subjects and healthy controls focusing on the anterior cingulate cortex (ACC), striatum, thalamus, and OFC and summarized the different and inconsistent results considering methodological factors [67] (Table 2). Specifically, some of these studies reported decreased N-acetylaspartate (NAA) resonance peaks (a measure of neuronal integrity) in the ACC and striatum of OCD patients. However, most studies found no significant differences in NAA resonance peaks between OCD patients and controls. Other studies reported, vice versa, decreased glutamate concentrations in the ACC and increased Glx (glutamate and glutamine) peaks in the OFC of OCD patients [57,67] (Table 2). Studies conducted with MRS revealed significantly lower NAA concentrations in the ACC without differing NAA concentrations by medication status [68]. A study with a combined approach of ^1^H-MRS and functional MRI (fMRI) in OCD patients compared to healthy subjects showed a reduction in NAA in the dorsal ACC of OCD subjects and a negative correlation between NAA and blood oxygen level-dependent activation during tasks [69], suggesting that multiple brain regions, including the ACC and frontal and temporal regions, could be involved in OCD pathophysiology [70]. Despite the absence of differences in caudate volumes between OCD patients and controls, the lower level of NAA in the patients’ left striatum body suggests a reduced neuronal density in this region [71]. Structural MRI data showed changes in the regional grey matter density in cortical and subcortical structures belonging to the CSTC circuit in OCD patients (reduced grey matter volume in the medial frontal gyrus, medial OFC, and left insulo-opercular region; increased grey matter volume bilaterally in the ventral part of the putamen and anterior cerebellum) [72]. The involvement of specific neurotransmitters in the pathophysiology of OCD is still unclear, but there are shreds of evidence suggesting serotonergic and dopaminergic abnormalities [73]. Concerning OCD associated with antipsychotic treatment, fMRI findings, attempting to elucidate differential SGA effects on the brain region of the CSTC, show a trend toward increased activation of the OFC—as well as of the left para-hippocampal gyrus, globus pallidus, and the right precentral gyrus—in clozapine/olanzapine-induced OCS schizophrenia patients during a response inhibition task [74] (Table 2), proposing the differential activation patterns that may reflect the pathogenic mechanisms in the development of OCS induced by SGA. Interestingly, in vivo ^1^H-MRS imaging showed that NAA in schizophrenia patients was significantly lower compared to controls in the left and right ACC regions [75]. These NAA findings may support neuronal loss in the ACC in schizophrenia [75], suggesting overlapping regions of dysfunction seen in OCD. Growing evidence implicates glutamatergic dysfunction in OCD [76] with relevance to glutamatergic cortico-striatal neuronal pathways, and their dysconnectivity may be considered a transdiagnostic signature in both schizophrenia and psychotic bipolar disorder [77]. The possibility of reduced Glx has been reported mainly in subgroups of OCD patients [67], especially those with mutations in genes affecting glutamatergic neurotransmission [78], or also related to the female gender [79]. A longitudinal ^1^H-MRS neuroimaging study conducted in drug-naïve children with OCD before and after 12 weeks of monotherapy with the SSRI paroxetine found significantly higher caudate Glx concentrations in patients compared to controls. Decreased Glx levels were reported after paroxetine treatment in OCD patients associated with an improvement in symptoms’ severity [65]. These findings suggest that Glx reductions in the caudate nucleus in pediatric OCD patients responsive to SSRI treatment could be modulated by serotonergic agents [65]. An alternative explanation provided by Rosenberg and colleagues about the possible inverse relationship between Glx levels in the ACC and caudate of OCD patients argues that reduced tonic glutamatergic firing in the ACC may predispose patients to increased stress-related phasic glutamate release in the caudate [80]. Multiple lines of evidence, mostly indirect, suggest that alterations in dopaminergic neurotransmission may contribute to the pathogenesis of OCD [73], data confirmed by the beneficial clinical effect that some OCD patients experience with the addition of antipsychotics to SSRI treatment [81,82]. A single photon emission computed tomography (SPECT) study provides in vivo evidence of significantly lower dopamine D2 receptor (D2R) binding in the left caudate nucleus in OCD patients compared to healthy controls, suggesting the direct involvement of the dopaminergic system in the pathophysiology of OCD [83] (Table 2). A positron emission tomography (PET) study evaluated the coexistence of serotonergic and dopaminergic dysfunction in OCD subjects [73] (Table 2). This study showed a significant reduction in the availability of [^11^C]MDL, a selective 5-HT_2A_ antagonist, in the polar, dorsolateral, and medial frontal cortex as well as in the parietal and temporal associative cortex of OCD patients, with a significant correlation between the availability of 5-HT_2A_ in the OFC and dorsolateral frontal cortex and the severity of clinical symptoms. In addition, the results showed a reduction in the uptake of [^11^C]Raclopride, a selective D2R antagonist, in the entire striatum, particularly in the ventral portion, possibly reflecting endogenous dopaminergic overactivity [73]. Abnormal spontaneous brain activity within cortico-striatal neural circuits has been observed in schizophrenia and OCD patients [84]. Resting-state functional connectivity (rsFC) indicates that the hippocampus and the left posterior cingulate cortex are shared regions exhibiting abnormal local spontaneous neural activity in schizophrenia and OCD patients. If the peculiar rsFC of the striatum between the caudate and the cerebellum reflects patients with schizophrenia, on the other hand, the increased rsFC of the left caudate between the right thalamus and the bilateral supplementary motor complex appears to be a potential hallmark of OCD patients [84] (Table 2). Wang and colleagues [85], using trait-based spatial statistics and probabilistic tractography to analyze the pattern of white matter abnormalities in schizo-obsessive comorbidity, found that these patients compared to healthy controls exhibited reduced fractional anisotropy and increased radial diffusivity in the right sagittal layer and left crescent of the fornix/stria terminalis (Table 2). In this context, an altered connection was shown in the default mode network, subcortical network, attention network, task control network, visual network, somatosensory network, and cerebellum in patients with schizo-obsessive comorbidity [85]. Neuroimaging studies have hypothesized that brain abnormalities in schizo-obsessive patients may be supported by an early onset of the illness [86]. Together, these results have led to extending the neurobiological interpretation of the obsessive-compulsive phenomenon beyond serotonergic neurotransmission and pointing to the investigation of dopaminergic and glutamatergic systems’ complementary interactions [57,87,88].

### 2.4. Pharmacological Studies in Schizophrenia Patients with OCS Comorbidity: The Putative Connection between Serotoninergic, Dopaminergic, and Glutamatergic Neurotransmission

Several studies have evaluated the occurrence of OCS in schizophrenia subjects treated with SGAs, both in terms of de novo occurrence and exacerbation of pre-existing OCS in clinically stable patients [90,91,92,93,94,95,96,97,98]. The use of SGA and the manifestation of OCS have been reported in previous studies [99,100] compared with selective anti-dopaminergic agents [95]. Schirmbeck and co-workers reported a reduction in OCS occurrence in schizophrenia patients treated with amisulpride, which mainly interacts with dopamine receptors, and the partial agonist of 5-HT_1A_ aripiprazole with neutral or anti-obsessive effects compared to clozapine and olanzapine, suggesting a serotonin imbalance in schizophrenia patients with OCS [95] (Table 3). The serotonergic antagonism of SGAs may lead to dysregulation between serotonergic and dopaminergic systems, contributing to the OCS [27]. The authors proposed that, before starting treatment with SGAs, it would be useful to assess specific neurocognitive domains, such as visuospatial learning and impulse inhibition performance domains commonly associated with frontostriatal and orbitofrontal functioning, that are valuable for the early detection of OCS SGA-induced in schizophrenia [95].

These observations are important as the authors suggested that the strong antagonism at serotonin receptors and simultaneous blockade action on dopaminergic receptors may constitute a key mechanism involved in the induction of OCS [27,74]. In this frame, other findings also showed that a total score above 70 on the positive and negative syndrome scale (PANSS) and the use of antidepressants predicted SGA-induced OCS in schizophrenia patients [97]. A causal relationship between the development of OCS and the use of SGA has not been conclusive, as OCS appears throughout the life of patients with schizophrenia [29]. Significant correlations of OCS severity with treatment duration, dose, and serum levels suggest a possible occurrence of clozapine-induced OCS. However, the putative causal interactions and neurobiological mechanisms underlying this pathogenetic process need further investigation [96]. Clozapine-induced OCS is not an uncommon side effect. Clozapine is believed to be implicated in the onset or exacerbation of already present OCS in patients with schizophrenia [25,95,101]. In this regard, a longitudinal study showed that obsessional thinking and hoarding behavior, but not compulsions, were significantly associated with the impact of clozapine treatment in schizophrenia patients with OCS [102] (Table 3). These side effects could be related to the higher plasma concentration of clozapine, which is handled with routine dose control [92]. In this context, a cross-sectional study comparing the prevalence and severity of OCS and OCD in 60 patients with schizophrenia treated with clozapine or haloperidol showed greater severity of OCS in patients using clozapine (*p* = 0.027) [97]. Although other authors found that clozapine treatment could be associated with a higher prevalence of OCS, especially in those receiving it for six months or more, they did not exclude the possibility that this association was related to the intrinsic aspect of the disease [93]. Of interest, several studies have reported that the combination with aripiprazole results in a marked improvement of OCS with or without further improvement of psychotic symptoms [94,103], suggesting an anti-obsessive effect of aripiprazole that is possibly associated with both dopamine and serotonin receptor partial agonism [94,104].

SSRIs have been found beneficial in some cases for treating OCS in schizophrenia and may be considered in augmentation with antipsychotics [29,105]. Some, albeit limited, advantages have been reported with the augmentation of antidepressants to clozapine, including clomipramine—a tricyclic antidepressant [106]—and SSRI, such as fluvoxamine [107], although with discordant results [108]. The SSRI treatment response in these patients is still controversial. Several studies supported the observation that SSRIs, especially fluvoxamine, are effective in schizophrenia patients with OCS [109,110]. Two case reports [111,112] reported the paradoxical action of fluvoxamine: the beneficial effect on OCS in schizophrenia on one side, and the increase in clozapine serum levels—via inhibition of CYP1A2 and 2C19 [111]—and thus the possible trigger action on the development of second-onset OCS. Dopamine and serotonin abnormalities have been demonstrated in patients with OCD [113] in treatment with SSRIs and antipsychotics, suggesting that dopamine receptor antagonism may further reduce the severity of OCS, particularly in those with tic disorder comorbidities [114]. Bloch and colleagues hypothesized that some forms of OCD are associated with a dysregulated dopaminergic function [114]. Several case reports concerning OCS treatment in schizophrenia [94,103,115,116,117,118,119,120] have shown some positive responses to lamotrigine, aripiprazole, paliperidone, amisulpride, and ziprasidone [121]. However, conclusive evidence supporting persistent OCS in schizophrenia treatment is scarce, and there is limited evidence on the beneficial effects of SSRIs, aripiprazole, mirtazapine, and cognitive behavioral therapy [122]. Cognitive behavioral therapy, as an adjunctive treatment to clozapine, was also reported as useful, particularly when drug treatment has been ineffective [123]. Even considering pharmacokinetic aspects, reports of effective vs. non-effective interventions—in some cases regarding the same molecules—in OCS schizophrenia patients seem strongly suggestive of more than one underlying physiopathological mechanism [121]. An open-label study using the glutamate antagonist riluzole showed significant anti-obsessive properties in treating refractory OCS [124] (Table 3), proposing glutamate-modulating drugs as an alternative add-on pharmacological strategy for refractory OCD [125]. Venkatasubramanian and co-workers postulated the role of a hyperglutamatergic state in OCD pathogenesis, in accordance with anatomical substrates such as the CSTC circuit, in which glutamate is the main excitatory neurotransmitter [59]. In line with these findings, in addition to acting on a serotonergic imbalance, pharmacological modulation of dopaminergic and glutamatergic systems may also be considered in OCS treatment, suggesting a connection between abnormalities of these neurotransmitters and their implication in the pathogenetic basis of OCS in schizophrenia [40,59].

**Table 3:** Antipsychotics’ anti/pro-obsessive effects based on receptor profile. D2R, dopamine D2 receptor; OCS, obsessive-compulsive symptoms.

Drug	Action on Neurotransmitter’s Pattern	Clinical Effects	Reference
Amisulpride	D2R antagonism	Reduction in OCS occurrence in schizophrenia patients	[95]
Aripiprazole	Serotonin and dopamine partial agonism	Neutral or anti-obsessive effects in patients treated with clozapine	[95,96,104]
Clozapine	Serotonin antagonism	Obsessional thinking and hoarding behavior, but not compulsions probably related to clozapine plasma concentration	[92,102]
Riluzole	Antiglutamatergic action	Anti-obsessive effects in treating refractory OCS	[124]

## 3. Schizophrenia and OCS: Current Pathophysiology Hypotheses and Relevance for Glutamate, Dopamine, Serotonin, and Their Interplay

The above-mentioned studies support the involvement of glutamatergic, dopaminergic, and serotoninergic neurotransmissions in both the occurrence and possible treatment options of OCS in schizophrenia patients. However, molecular studies on this complex interaction are still lacking. Evidence from preclinical studies may provide some key suggestions on the interplay of these three neurotransmitters in OCS overlapping schizophrenia symptoms. OCS, when part of OCD, responds selectively to SSRIs; however, despite the efficacy of SSRIs in the treatment of OCD, little and sparse evidence underlying serotonin deficit or variants of serotonergic genes in the developing of disease symptomatology is available [126,127,128,129]. On the other hand, growing evidence also points to dopamine and glutamate involvement in the biological basis of OCS, considering the possible treatment response to D2R antagonists in those cases difficult to treat. Dopamine plays a role in stereotypic behavior in preclinical settings [130]. It is involved in cognitive and affective processes, including reward processing [131], which may shape the “cognitive and reward side” of OCS. Regarding reward and anxiolytic effects, a recent preclinical study focused on the role of neurotensin and its regulation by D1-like and D2-like receptors antagonists (i.e., SCH23390 and sulpiride) that are able to revert these effects [132]. Despite no evidence confirming its direct link with OCD or OCS, neurotensin could have a role in neurotoxicity by activating NMDAR, resulting in further activation of glutamatergic loops that are hyperactive in OCD [133,134]. Dopamine is a key player in schizophrenia physiopathology, and its alteration is considered responsible for several clinical manifestations. Growing evidence regarding genetic variants in catecholaminergic genes and decreased striatal D2R levels reports a link between TRS and OCD [129,135]. The glutamatergic system, as well, appears to be implicated in the neurocircuits relevant for OCS as reported by both cerebrospinal fluid and MRS studies that reveal alterations in glutamatergic metabolites [67,136] and genetic findings concerning GRIN2B and DLGAP1 [19,137], which are also strongly associated with schizophrenia [138,139]. Our interest is related to possible convergence points on neurotransmitters involved in the emergence of OCS in schizophrenia. In this regard, glutamate implication in orchestrating schizophrenia symptoms is demonstrated by several both clinical and preclinical studies [140,141,142] that have led to the formulation of the “glutamate hypothesis of schizophrenia” related to NMDAR hypofunction [143]. On the other hand, studies on the serotoninergic system related to hallucinogenic drugs, such as lysergic acid diethylamide [144], as well as the observation of antipsychotic effects of serotonin–dopamine antagonists, such as clozapine and risperidone, have driven a growing interest in this field as a possible pathophysiological target in schizophrenia [145]. Growing evidence hypothesizes that psychosis involves neural networks beyond the canonical mesolimbic dopaminergic pathway [146], including the serotonin and glutamate systems [147]. Physical and functional interactions between serotonin–glutamate and serotonin–dopamine signaling have been hypothesized to be involved in the pathophysiology of psychosis, relevant for antipsychotic treatment [148], synaptic plasticity and functional connectivity [149].

Several clinical and preclinical studies focus their attention on dopamine–serotonin-glutamate neurotransmission convergence points. Intracellular molecules, as well as receptor heterodimerization, are responsible for integrating different types of signaling in response to extracellular stimuli and drugs [146].

The cyclic adenosine monophosphate (cAMP)-regulated phosphoprotein, 32 kDa (DARPP-32), is a common point of convergence of glutamate and dopamine neurotransmission. It is localized in the mid-spiny neurons of the striatum [150,151] that represent nodal relays in the CSTC circuit, which controls complex behaviors such as habits [152,153]. DARPP-32 is phosphorylated and acts as an information integrator between the cortex and the basal ganglia representing a possible crosstalk site between dopamine and glutamate transmission [154,155,156]. DARPP-32 has been linked to schizophrenia molecular pathophysiology: (i) it is found reduced in animal models of psychosis *and postmortem studies* on schizophrenia brains [157,158,159]; (ii) its phosphorylation is modulated and increased by antipsychotic therapies such as haloperidol [160,161]. It was defined as a hub, in striatal medium spiny neurons, for multiple kinases (including protein kinase A—PKA) and phosphatases (including protein phosphatase 1—PP1), via both D1-PKA and calcineurin-glutamate [162,163,164,165]. Another possible functional intersection between dopamine and glutamate is represented by STriatal-Enriched protein tyrosine Phosphatase (STEP) in the postsynapses of the medium spiny neurons, which is phosphorylated and subsequently inhibited by both D1R (via PKA) and NMDAR (via ubiquitination and degradation) neurotransmission [166,167]. This effect is responsible for cognitive performance amelioration as demonstrated in STEP KO mice and increased phosphorylation of extracellular signal-regulated kinases (ERK), Fyn, and GluN2B, possible druggable targets in neuropsychiatric disorders treatment [168]. Dopamine, glutamate, and serotonin interplay is modulated by drugs, including clozapine, thanks to multiple heterodimers activating pathways different from the canonical pathways [169]. Bioluminescence energy transfer, fluorescence resonance energy transfer, and in vivo Proximity Ligation Assay studies demonstrated dimerization of 5-HT2Rs with D2Rs in rat striatum [170,171,172] with different responses to dopamine and serotonin agents, resulting in an asymmetrical and ligand-dependent cross-regulation [173,174]. Intriguingly, in an in vitro study, a mutant for 5-HT_2A_ associated with clozapine resistance in humans, exhibit a reduced capacity to form this type of heterodimers [175]. 5-HT_2A_ is also able to constitute heterodimers with metabotropic glutamate receptor 2 (mGluR2), resulting in the potentiation of mGluR2-G_i_ signaling and inhibition of 5-HT_2A_-G_q_ signaling, restored by clozapine [176]. The presence of D1R-D2R heterodimers is increased in the globus pallidus of schizophrenia patients and seems to be responsible for promoting grooming behavior and α-amino-3-hydroxy-5-methyl-4-isoxazolepropionic acid receptor (AMPAR)’s GluR1 phosphorylation [177,178]. Finally, dopamine receptors are also involved in crosstalk with NMDAR pyramidal and striatal neurons and interneurons: D1R activation is responsible for enhanced NMDAR-mediated Ca^2+^ release, whereas D2R activation inhibits NMDAR neurotransmission that could be implicated in the hypoglutamatergic transmission reported in schizophrenia and in restoring cortical–striatal connectivity and synaptic plasticity, relevant for cognitive processes [151,179,180].

Taken together, these findings may partially emphasize the complexity of the interplay between dopamine, glutamate, and serotonin and could allow OCS to be read in the context of schizophrenia as a dysregulation of the circuits canonically involved in the disorder, with an imbalance that could be responsible for the simultaneous occurrence of both compulsive and psychotic symptoms as a psychopathological and endophenotypic continuum.

### 3.1. Neural Circuits

#### 3.1.1. Glutamate and OCS in Schizophrenia

The canonical hypothesis of OCS is based on the dysregulation of the CSTC loops [181] that contain overactivated glutamatergic input originating from cortical-efferent fibers targeting the basal ganglia. Within this circuit, two different pathways have been considered: the direct pathway, in which excitatory glutamatergic signal projects to the striatum, inhibiting the internal part of the globus pallidus via γ-aminobutyric acid (GABA) with subsequent disinhibition of the thalamus, resulting in an increased excitatory effect on the cortex; and the indirect pathway, from the cortex to the external part of the globus pallidus and the subthalamic nucleus that is responsible for an excitatory signal to the internal part of the globus pallidus [182,183]. The functional implications of the direct pathway are related to a self-reinforcing positive feedback loop that contributes to the initiation and continuation of behaviors. The indirect pathway provides for the inhibition of behaviors and switching between behaviors. It may be concluded that human behavior depends on a flexible balance between initiation and inhibition [181,184].

However, based on the assumption that in the ventral part of the prefrontal cortex (PFC) a difference between ventral and dorsal circuits exists in terms of functional meaning, new and more detailed modeling is proposed to explain the neural circuits involved in the compulsivity. In this regard, two new circuits are defined: ventral–emotional and dorsal–cognitive pathways. The two circuits have different timings of neurodevelopment, and it could be related to their function; ventromedial areas of the PFC (i.e., OFC, dorsomedial prefrontal cortex, and ACC) are involved in the control of emotional behaviors and develop relatively early, whereas lateral regions of the PFC (i.e., DLPFC and VLPFC) develop later and are implicated in executive functions [185].

Coherent with the above-mentioned evidence, Milad and Rauch [185] have suggested three different circuits regulated by cortical glutamate excitatory outputs:

- The affective circuit, from ventromedial-PFC and ACC to the nucleus accumbens (NAc) and the thalamus, relevant for affective and reward processing.
- The dorsal cognitive circuit, from the dorsolateral-PFC to the caudate nucleus and the thalamus, relevant for executive functions.
- The ventral cognitive circuit, from the anterolateral OFC to the anterior part of the putamen and thalamus, relevant for motor preparation and response inhibition [185].

On the other hand, the change in nosographic categorization of OCD with the shift from “anxiety disorders” to “obsessive-compulsive and related disorders” [186] has suggested the involvement of another circuit, the sensorimotor circuit, that includes the (pre)motor cortex, posterior part of the putamen and thalamus and is responsible for mediating the automatic response and the transition from goal-directed to habitual behaviors, reducing dependence on striatal dopaminergic projections [187,188]. In murine animal models, the sensorimotor loop is involved in the interplay between somatosensory and motor cortical regions, and the dorsolateral striatum seems to be implicated in the division of motor patterns during habit formation and imprinting [189,190,191]. Disruption of these connections impairs the learning and execution of motor sequences and stimulus-response tasks, as well as the usual response in instrumental tasks that follows earlier goal-directed performance [189,192], cooperating with the emergence of compulsivity via the dopaminergic projections at the striatal level [193].

Growing evidence also points to the amygdala and limbic system activation that appears to be associated with the experience of OCS [70,194,195,196,197,198] and reactivity to emotional faces in OCD [199,200], with a parallel increase in amygdala–fronto-parietal network connectivity [199]. Neuroplasticity processes are responsible for different loop modulations during the whole lifespan, explaining the differences in the symptomatology of OCD; Geller and co-workers reported a prevalence of movement disorders such as tics, choreiform, or other involuntary, abnormal hyperkinetic movements in childhood and subsequent involvement of the sensorimotor CSTC circuit [201]. However, an increase in ventral striatal volumes and neurodegeneration in the limbic brain areas, probably related to chronic compulsivity, anxiety, and compensatory processes, was observed in adults [72,202].

On the other hand, a disrupted CSTC loop is also involved in schizophrenia dysfunction, as demonstrated in a recent neuroimaging study [203]. Several studies demonstrated a double pattern of dysconnectivity related to the thalamus: a high thalamic coupling with sensorimotor areas and a lower one with associative regions [204,205,206]. However, the alteration in connectivity between associative regions was found to be more frequent in schizophrenia than in OCD patients [203], but the coexistence of both dysfunctions could be coherent with OCS in schizophrenia.

Network connectivity has contributed to explore the differential activation of ACC and OFC in schizophrenia and OCD. A recent study found that projections from the right anterior insula to the right medial OFC are reduced, whereas those between the right anterior insula and left dorsal ACC are increased in the OCD group with poor insight [207]. On the other hand, an impairment in the right anterior insula-OFC connections is also reported in schizophrenia patients with insight [207], suggesting that connections between these brain regions could be significant neural correlates of insight in OCD and schizophrenia [208]. Noteworthy, the striatum represents the input nucleus of the basal ganglia, receiving excitatory afferents from both the cerebral cortex and thalamus, and acts as a critical structure for response inhibition driven by dopaminergic neuromodulation [209]. The dysfunction of response inhibition in cognitive control is strongly linked to obsessive-compulsive behavior. A significant positive association between dopamine synthesis and inhibition-related neural activity in the caudate nucleus mediated by striatal glutamate concentration has been reported in humans [210,211]. Glutamate and dopamine systems interact at multiple levels, and their signaling interplays both in cortical and subcortical structures, modulating neuroanatomical substrates relevant for the pathophysiology of psychotic disorders [212].

#### 3.1.2. Dopamine and Compulsions: Relevance for OCS in Schizophrenia

Szechtman H. and colleagues [213] investigated the effects of quinpirole, a D2R agonist, on rat behavior. They found that acute administration of this compound has a dose-dependent effect on locomotor activity. At low doses, it is responsible for a decrease in locomotor activity, while at higher doses, it causes an increase [214]. These effects are probably related to the activation of the presynaptic D2R with high affinity and the postsynaptic D2R with low affinity, respectively [215]. Interestingly, repeated administration of quinpirole leads to a gradual and sustained increase in locomotion, similar to the locomotor sensitization mediated by psychostimulants [216,217], and subsequently to the development of stereotypic behavior. These observations have led to the assumption that rats treated repeatedly with the dopamine D2 agonist quinpirole may represent a validated model for OCD [218,219,220]. Specifically, compulsive control [221], behavioral inflexibility, and compulsive chewing [222] characterize the behavior of rats after quinpirinole administration.

Overall, these data suggest that D2R is a key component in midbrain dopamine pathways with regard to locomotor sensitization and compulsivity [223], likely due to a subsequent imbalance between D1R/D2R [224]. Behavioral sensitization is associated with adjustments in the reward/motivation circuit. Rats treated with quinpirole exhibit a lower dopaminergic tone in the NAc, consisting of a reduction in basal firing [225], and an increase in tonic and phasic dopamine release [226], resulting in hypodopaminergic midbrain circuits. On the other hand, a decrease in both tonic and phasic firing of dopamine neurons in the ventral tegmental area was demonstrated in rats treated with quinpirinole [227], which could be another cause of a decrease in the overall activity of dopamine neurons in addition to the first [223]. These findings are also consistent with the observations that rats treated with quinpirole exhibit an increase in the binding of D2R and in its affinity state [213,228].

Other evidence suggests a hyperdopaminergic state in the ventral striatum that may be particularly relevant to OCS because of its involvement in excessive appraisal or the choice of appealing behavior [229]. In accordance with these findings, dopamine indirect agonists, like amphetamine, are triggers of repetitive behavior [230,231]. Hyperactivation of dopaminergic mesolimbic pathways is the widely accepted working hypothesis for positive symptoms in schizophrenia, which assumes an association between the occurrence of OCS and the lifespan of patients affected by schizophrenia. On the other hand, evidence is accumulating in favor of a more complex interplay between OCS and dopamine. For example, there is evidence that dopamine antagonists may exacerbate symptoms, and that there is both increased and decreased binding to the dopamine transporter [232]. These data should be better reconciled with observations that anti-D1R and anti-D2R antibodies are common in patients with OCS [233,234]. In schizophrenia patients, shifting response sentences associated with perseverative responses are frequently impaired, which is probably due to task-inappropriate D2R-mediated reselection of a previously activated cortico-striatal process [235] or as a result of low D2R stimulation [236]. In conclusion, the dysregulated signal in the dopamine system is strongly associated with both OCS and psychotic symptoms, presumably due to an impaired downstream dopamine regulatory pathway.

#### 3.1.3. Serotonin and the Occurrence of Obsessive Symptoms in Schizophrenia

The observation that patients affected by OCD are responsive to SSRIs led to the assumption that the serotonin system could be involved in the neurobiological basis of the disease. Although SSRI demonstrates efficacy, there is surprisingly little evidence of a serotonin deficit as a primary causal role in OCD’s biological underpinnings [126]. Differences in serotonin levels and metabolites are reported in the literature, particularly regarding patients with familiarity [126]. A reduction in the availability of SERT in the thalamus and midbrain was reported in a SPECT study [237] in OCD patients, and low levels are associated with increased symptom severity [238,239]. SSRIs are probably responsible for increased serotonin levels in the limbic areas and basal ganglia reducing obsessions [240]. A preclinical study investigated the serotonin receptor subtype involved in obsessive-compulsive-like behavior, identifying the orbitofrontal 5-HT_1B_ receptor as responsible for inducing OCD-like symptoms in mice and concluding that SSRIs reduced OCD-like behavior by desensitizing these receptors [241]. The same mechanism of action is related to the 5-HT_1A_ receptor [242,243], but the therapeutic effect of SSRI seems to involve the downregulation of serotonin receptors [244]. Although the serotonin system may play a role in both the treatment and etiology of OCD, the specific neurobiological basis remains unclear [244]. Meanwhile, serotonin circuits represent a player in the neurobiology of schizophrenia, considering the evidence that hallucinogenic drugs are responsible for inducing psychotic symptoms that are improved after serotoninergic agent administration, such as clozapine [144,145]. These results lead to the hypothesis that serotonin receptor localization and imbalanced expression could partly explain OCS in psychosis and may be related to both drug treatment and neurobiological dysregulation.

### 3.2. Neurotransmission Overlapping: Relevance for OCS in Schizophrenia

While serotoninergic neurons of the dorsal raphe receive input from the PFC whose function is associated with motivation in a mice animal model [245], subcortical inputs and connection with direct and indirect circuits are responsible for the *s*election of goal-directed behavior evaluated in a recent preclinical study in a rat model [246]. In addition, the dorsal raphe appears to be involved in self-stimulation, which strongly promotes behavior via dopaminergic neurotransmission [247]. In contrast, the punishment could be triggered by serotonin or by the reduction in dopamine [248]. Several preclinical studies have focused on the timing of serotonin and dopamine release, concluding that these neurotransmitters are able to transiently modulate behavior, resulting in transient or sustained effects depending on different brain regions, as well as synaptic, cellular, and epigenetic mechanisms [249].

A structural and functional interplay between the 5HT_2A_ receptor and mGluR2 may result in the formation of heteromeric complexes relevant to the mechanism of action of hallucinogens as well as for antipsychotics [250]. In addition, the imbalance between the 5HT_2A_/G_q11_ and mGluR2/G_i/o_ pathways is responsible for the different effects of molecules at 5HT_2A_/mGluR2 that act as pro- or antipsychotic drugs [250]. Serotonin-dependent glutamate release is also modulated by glutamate itself via mGluR2 inhibition at the presynaptic level in pyramidal cells from the PFC [251]. These two types of receptors interact at the epigenetic level; chronic SGA administration is associated with the downregulation of mGluR2 in mice frontal cortex [252] via 5HT_2A_ and nuclear factor kappa-light-chain-enhancer of activated B cells that are involved in the in regulation of histone deacetylase 2 [252,253].

Glutamate neurotransmission is also under reciprocal dopaminergic regulation, probably via NMDAR, considering that antagonists at these sites (i.e., phencyclidine and (+)-5-methyl-10,11-dihydroxy-5H-dibenzo(a,d)cyclohepten-5,-10-imine; dizocilpine-MK-801) are responsible for increased dopamine function and metabolism [254,255,256]. On the other hand, glycine, a positive allosteric modulator of NMDAR, induces an increase in dopamine release [257,258]. The same results are reported after AMPAR and kainate agonist exposure in rat striatal slices reinforced NMDAR presynaptic activation [259].

The interplay among these neurotransmitter systems remains to be investigated and is likely to be closely related to cell types, brain regions, timing of stimulation, and phase of psychiatric disorder. From this perspective, OCS in schizophrenia could occur as an imbalance of neurotransmission or even cell typology during the disorder or the neurodevelopmental process.

## 4. Postsynaptic Density: Implication for the Transdiagnostic Dimensions of Compulsions

The synaptic interactions between dopamine and glutamate signaling can take place at several levels of the synaptic structure, including the postsynaptic density (PSD). The PSD is detectable as a dense electronic microscopy disk-shaped structure beneath the postsynaptic membrane [260,261]. It is conceptualized as a network of proteins that includes receptors, scaffolds, adaptors, and cytoskeleton proteins organized in a triple layer, deputed to shape the architecture of the dendritic spine and propagate transduction signals from the cell surface to inside the cell. It appears as specialized ultrastructure localized at the glutamatergic excitatory synapses membrane and is involved in several functions critical for dopamine and glutamate-dependent synaptic plasticity processes [211]. Dysfunction of the PSD protein network may lead to severe impairment of calcium-dependent signaling with a functional and structural disruption of synapsis, which is modulated by antipsychotics that emphasize the role of the dopamine–glutamate aberrations implicated in the pathogenesis of psychotic disorders [260,262,263,264]. PSD scaffolding proteins are critically involved in dopamine–glutamate interplay, acting as a key agent in many psychiatric disorders related to altered synaptic plasticity [212] such as OCD and schizophrenia.

NMDAR represents the core of PSD, together with AMPAR and mGluR type I (mGluR 1 and mGluR 5) [265]; the localization of these receptors at the postsynaptic membrane is mediated directly or indirectly by physical interaction with a large number of scaffolding/adaptor proteins, including PSD-95, guanylate kinase-associated protein (GKAP), glutamate receptor-interacting protein (GRIP), proteins interacting with C kinase-1 (PICK1), Shank, and Homer, and is modulated, among others, by antipsychotic treatment [266,267]. Through these proteins, neurotransmitter signaling is transduced to molecules such as calmodulin-dependent protein kinase II (CAMKII) or the GTPases Ras, Rho, Rac, and ADP ribosylation factors (Arf) [268]. PSD, as a signalosome and connectome, is responsible for multiple activities of neurons, including spine formation, maturation, and the enlargement of dendritic spines. Several PSD proteins are involved in schizophrenia neurobiology, including Shank, which forms a polymeric network complex with Homer tetramers and Shank multimers [269,270]. PSD-95 is essential for dendritic spine morphogenesis, stabilization, and trafficking of glutamatergic receptors [271,272,273]. Homer1 is involved in working memory and executive functions [274,275,276], exists in multiple isoforms, and contributes to dendrite morphology and axon guidance through tetramer formation regulated by Homer1a levels [270]. It is well established that PSD molecules are modulated by psychotropic drugs including antidepressants, antipsychotics and mood stabilizers alone or in combination [277,278,279,280].

**Figure 1:**
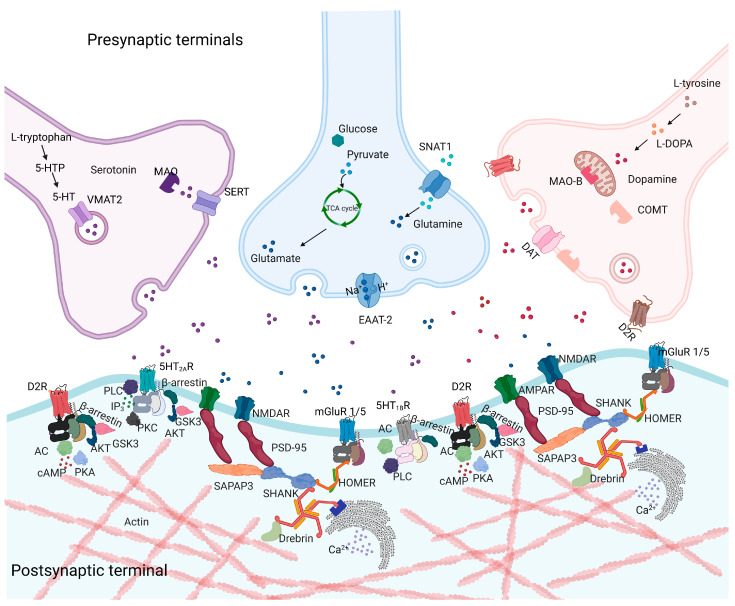
Diagrammatic and comprehensive overview of glutamate–dopamine–serotonin interaction at the postsynaptic density site. 5HT_1B_R, 5-hydroxytryptamine receptor 1B; VMAT, vesicular monoamine transporter; 5-HT, 5-hydroxytryptamine; 5HT_2A_R, 5-hydroxytryptamine receptor 2a; SERT, serotonin transporter; EAAT-2, excitatory amino acid transporters; DAT, dopamine transporter; D2R, dopamine 2 receptor; AC, adenylyl cyclase, cAMP, cyclic adenosine monophosphate; GSK3, glycogen synthase kinase-3 beta; PLC, phospholipase C; IP_3,_ phosphatidylInositol 3-Kinas; PKC, protein kinase C; PSD-95, postsynaptic density protein 95; SAPAP3, SAP90/PSD-95-associated protein; mGluR1/5, metabotropic glutamate receptor 1/5; MAO, monoamine oxidases; MAO-B, Monoamine oxidase B; 5-HTP, 5-Hydroxytryptophan; SNAT, sodium neutral amino acid transporter; L-DOPA, l-3,4-dihydroxyphenylalanine. Created with BioRender.com, accessed on 28 February 2023.

No direct findings are available regarding the above-mentioned proteins for the neurobiological basis of OCD except for SAPAP3, a PSD protein, that could represent a possible mechanism shared by OCD and schizophrenia [281,282,283,284] (Figure 1).

### 4.1. SAPAP Proteins: Relevance for OCS in Schizophrenia

The SAPAP family includes four postsynaptic scaffold proteins, resulting from different splicing, that are highly concentrated at the PSD and relevant for maintaining the structure and functioning of synapsis [285] (Figure 1). Due to their differential expression in several brain regions [286,287], the members of the SAPAP family cooperate in multiple physiological roles.

The SAPAP family is a group of proteins characterized by direct interaction with PSD-95 (via the GK domain) [288] and Shank (via the PDZ domain) [289,290] proteins, primarily involved in schizophrenia [291,292]. As a result of their interaction with PSD-95, SAPAP proteins indirectly interact with NR2A and NR2B subunits of NMDARs, Shaker-type K+ channels [293,294], neuroligin [295,296], and AMPAR via stargazing [297]. Furthermore, the connection of SAPAP with Shank proteins is relevant for the crossplay with mGluR type I via Homer proteins [298], and the actin cytoskeleton [289,299], contributing to the formation of the PSD-95/SAPAP/SHANK complex that is central for the plasticity of the glutamatergic synapses [288].

#### SAPAP3 and Its Involvement in Synaptic Plasticity and Repetitive Behavior

During the postnatal period, the SAPAP isoforms are expressed differently in the neurodevelopmental process. The spatiotemporal expression pattern of the isoform SAPAP3 is characterized by a peak expression observed around 2–3 weeks after birth in the cortex, striatum, and thalamus [287,300]. This isoform is expressed at the dendritic level in the striatum, several thalamic nuclei, layers 1–3 of the cortex, and moderately in the amygdala [42,286,287,301,302]. SAPAP3 is the only family member highly localized in dendrites [287,300], suggesting its unique role in synaptic plasticity. SAPAP3 tends to be predominantly localized at the corticostriatal excitatory synapses, including glutamatergic and cholinergic synapses, but not at the inhibitory synapses [42,303]. Its deletion in KO mice results in pathological grooming and anxiety-like behavior [304], data not yet confirmed in human studies [305]. CaMKII phosphorylation of SAPAP is nodal for the PSD-95-SAPAP and SAPAP-DLC interactions relevant for synaptogenesis processes [306,307]. Interestingly, it has been demonstrated that antidepressants widely used to treat OCD symptoms may affect phosphorylation l of alpha-CamKII [308]. Consistent with these findings, in vivo studies highlighted the potential role of SAPAPs in synaptic development; the homozygous deletion of SAPAP3 in mice showed an increase in NR2B subunit levels of NMDAR, which represent the “juvenile” subunit, whereas the “adult” subunit NR2A was reduced [42]. In the same animal model, a reduction in AMPAR subunits [42] with a subsequent decrease in AMPAR-mediated synaptic transmission relevant for OCD-like behavior was detected, suggesting a possible role in the formation of immature corticostriatal synapses and deficits in corticostriatal synaptic function [303]. SAPAP3 KO mice also exhibit a reduction in thickness of the PSD “dense layer” (composed of receptors and scaffolding proteins) but not in the PSD fringe “light layer” (composed of trafficking proteins and the actin cytoskeleton) of corticostriatal synapses with no changes in spine density in adult and young adult mice, indicating that spiny formation is not affected by SAPAP deletion [42].

SAPAP3 is also involved in monoaminergic system modulation. SAPAP3 KO mice exhibit an upregulation of the serotonin metabolite 5-hydroxyindoleacetic acid and the 5-hydroxyindoleacetic acid/serotonin ratio in all cortical and striatal regions [309] and response to SSRI treatment [42]. In a recent preclinical study, chronic clozapine treatment was found to induce obsessive-compulsive-like symptoms in SAPAP3 KO mice earlier than wild-type mice, and this effect was reverted by fluoxetine, supposing clozapine–gene interaction and the involvement of serotonin transmission in the emergence of OCS [310]. In addition, an increased dihydroxyphenylacetic acid/dopamine ratio is reported in the lateral OFC and the homovanillic acid/dopamine ratio was found to increase both in the lateral and medial OFC [309]. Alterations in dopamine receptor density are reported in the NAc of SAPAP3 KO mice. Taken together, these data underline the effects of SAPAP3 on the three different neurotransmitters, indicating its role in the glutamate–monoamine interplay and representing a neurobiological underpinning for OCS in schizophrenia [212,311].

## 5. OCS in Schizophrenia, and Brain Neuromodulation in the Context of Dopamine–Glutamate–Serotonin Interaction

Emerging evidence points to neuromodulation techniques, including repetitive transcranial magnetic stimulation (rTMS) and transcranial direct current stimulation (tDCS), as promising methods for the treatment of both schizophrenia and OCD [312,313,314].

The rTMS and tDCS, each with its own specificities, are both techniques that are well tolerated and relatively feasible even in complex patients. The contraindications are few, (e.g., uncontrolled epilepsy, the presence of skull discontinuities, metallic bodies in the brain, or active medical devices), and there is evidence of safety in all age groups, with specific safety guidelines being available [315,316]. Even if no coded criteria are available to specifically qualify a patient with schizophrenia and/or OCD for rTMS and/or tDCS, it could be of interest to include brain neuromodulation techniques in the putative therapeutic armamentarium of OCS in schizophrenia based on the following considerations:

(a) the impact of both techniques of neuromodulation on multiple neurotransmitter systems, including serotonin, dopamine, and glutamate, in preclinical modeling [317,318]; (b) the bidirectional reciprocal effect of SERT inhibition by SSRIs and D2R antagonist (sulpiride) [319,320]; (c) the potential effects of neuromodulation on glutamatergic PSD as demonstrated in preclinical modeling [321].

Evidence has accumulated on the possible efficacy of brain stimulation techniques both for positive and negative symptoms of schizophrenia, as well as for the potential improvement of neurocognition. Regarding positive symptoms, the effectiveness of inhibitory stimulation—cathodal tDCS and low-frequency repetitive rTMS (LF-rTMS)—has been demonstrated for the treatment of auditory hallucinations, using as a target the left temporo-parietal junction, a brain area that results in hyperactivity during auditory hallucinations [322,323]. Regarding negative and cognitive symptoms, encouraging results have been obtained using excitatory protocols—anodal tDCS and high-frequency repetitive rTMS (HF-rTMS)—targeting the left DLPFC [324]. The stimulation of this area in schizophrenia patients may ameliorate cognitive deficits, such as working memory, as well as non-cognitive symptoms of schizophrenia, including auditory hallucinations and disorganization, with an effect lasting up to a month from the end of treatment [325]. Based on these observations, recent evidence has focused on the combination of excitatory stimulation over the left DLPFC with the cognitive remediation protocols commonly used in patients with schizophrenia, to boost physical or cognitive impairment in stroke [324]. Encouraging results were obtained targeting the cortical areas of the CSTC loop involved in the OCD pathophysiology, especially the complex pre-supplementary motor area (pre-SMA)/ACC, the DLPFC, and the OFC. Consistent with the neuroimaging evidence of hyperfunction in these areas in OCD, the cortical inhibition induced by cathodal tDCS protocols has proven its efficacy [326,327]. Moreover, in LF-rTMS studies, the treatment was also shown to normalize the altered functional connectivity among the regions included in the OCD circuits [328]. Future therapeutic strategies based on brain stimulation techniques or protocols could be implemented as a treatment for patients with schizophrenia and OCD, considering the following: (1) the efficacy on schizophrenia symptoms or OCS; (2) the evidence derived by neuromodulation targeting at the same time the brain areas effective for both OCD and schizophrenia, even if the protocols are not considered beneficial in the two conditions separately; and (3) the activity targeting a neuropsychological function known to be impaired in both disorders (e.g., executive functions) [35]. In this case, the combination with cognitive training appears very promising, especially considering that schizophrenia patients with OCS display more severe neuropsychological impairment than both schizophrenia without OCS and OCD patients [329]. In summary, based on the encouraging results of a few studies both in schizophrenia and OCD, including patients that do not properly respond to the respective pharmacological treatments, more trials for TRS patients with significant OCS are warranted before making any further conclusions on neuromodulation in this difficult-to-treat condition.

## 6. Discussion

The co-occurrence of OCS in schizophrenia is not a rare condition, making even more complex both the behavioral manifestations and the outcome of schizophrenia, as well as contributing to the TRS condition, characterized by a severe clinical complication affecting cognition and functioning [330]. Therefore, there is a need to better understand the neurobiological basis of schizophrenia with OCS and its relationship with TRS. D2R occupancy, the main mechanism of action shared by all the available antipsychotics, may trigger multiple trans-synaptic effects strongly associated with dopamine–glutamate–serotonin interaction and impact brain circuits also implicated in the emergence of OCS. This triple interaction, possibly modulated by antipsychotics, may take place at different levels. At the first level, this is represented by the macro-circuits of brain regions believed to be involved, and partially overlap in the pathophysiology of both OCD and schizophrenia, including the OFC, temporal poles, and the cingulate cortex [331]. The second level of the interaction involves the microdomains of the synapse, which include at the neuronal membrane, the physical and functional cooperation between receptors such as D1R-NMDAR [332], 5HT_2_-mGluR [333], 5HT_2A_, and D2R, as demonstrated by multiple in vitro techniques, in particular fluorescence resonance energy transfer [172]. Finally, a third level, the effect of the dopamine, glutamate, and serotonin interactions and their changes after antipsychotic treatment could be positioned at the core of the dendritic spine, the PSD [334], especially at the glutamatergic synapse [211,266,335].

Despite the findings on dopamine–glutamate–serotonin interplay in schizophrenia and OCD, the conceptualization of its prominent role has unveiled several limitations that should be acknowledged. First, beyond the three major neurotransmitters here addressed and their reciprocal interactions, the proposed framework is a reductionism by itself. Other neurotransmitters, such as norepinephrine [336], GABA [337], opioids [338], and acetylcholine [339] for each of whom evidence in schizophrenia and OCD pathophysiology is available [126], could have been included in the complex platform of multiple neurotransmitters’ interactions putatively underpinning OCS in schizophrenia and its contribution to TRS. Second, both schizophrenia and OCD have been considered at least in part neurodevelopmental disorders; the dysregulation of the neurotransmitters therein addressed could be caused by very different aetiologies that may impact the neurodevelopment at an early (i.e., prenatal) or late (i.e., adolescence or young adulthood) stage, and therefore be only the tip of the iceberg in the overall pathophysiology of schizophrenia with OCS, as well as in their contribution to TRS [340,341,342]. Third, genetic and genomic findings have already shown a significant possibility of gene overlapping that may contribute to the presence of OCS in schizophrenia [21]. However, estimates of family-based genetic, shared and non-shared environmental influences, as well as SNP heritability, need to be investigated more deeply for significantly overlapping phenotypes in schizophrenia with OCS [343].

## 7. Conclusions

The presence of OCS in schizophrenia is a relevant clinical challenge whose neurobiological underpinnings remain for the most part obscure; however, findings from genetics, imaging, and preclinical modeling are emerging. The interaction between dopamine, serotonin, and glutamate may represent a possible molecular scalpel to better identify the molecular pathophysiology and putative strategies of this complex clinical condition. Nevertheless, the involvement and fine tuning of synaptic proteins especially at the glutamatergic dendritic spine may represent a novel possibility to explore the biological basis of this clinical manifestation and must be considered for further investigation.

## Figures and Tables

**Table 1:** Neurotransmitter pathway genetic susceptibility: obsessive-compulsive and psychosis dimension. SLC1A1, solute carrier family; EAAC1, excitatory amino acid carrier 1; DLGAP3, disc large associated protein 3; SAPAP3, SAP90/PSD-95-Associated Protein 3; Val, Valine; Met, Metionine; GRIN2B, glutamate ionotropic receptor NMDA type subunit 2B; COMT, catechol-O-methyltransferase; 5HTR, 5-hydroxytryptamine receptors; 5-HT_2A_, 5-hydroxytryptamine receptors type 2A; OCS, obsessive-compulsive symptoms.

Neurotransmitter	Gene	Protein	Genetic Variant	Functional Meaning	Reference
Glutamate	SLC1A1	EAAC1	rs2228622–rs3780413–rs3780412	Susceptibility of atypical antipsychotics-induced obsessive-compulsive symptoms	[40]
	DLGAP3	SAPAP3	rs7525948	The interaction with rs2228622 polymorphism of the SLC1A1 gene increases the risk of developing atypical antipsychotics-induced obsessive-compulsive symptoms	[41]
	SLC1A1 and GRIN2B	EAAC1 and NR2B	rs2228622 and rs890	Susceptibility to OCS in patients treated with clozapine	[58]
Dopamine	COMT	COMT	Val^158^/Val^158^ Val^158^/Met^158^ Met^158^/Met^158^	Met^158^/Met^158^ appears to act in a recessive manner and increase the susceptibility to developing OCD	[44]
Serotonin	5HTR	5-HT_2A_	rs6311-rs6313	Susceptibility to OCD	[46]

**Table 2:** Neuroimaging studies in schizophrenia, obsessive-compulsive disorder, and schizo-obsessive patients. ^1^H-MRS, in vivo proton magnetic resonance spectroscopy; SPECT, single-photon emission computed tomography; PET, photon emission tomography; fMRI, functional magnetic resonance imaging; OCD, obsessive-compulsive disorder; ACC, anterior cingulate cortex; CSTC, cortico-basal ganglia-thalamo-cortical; D2R, dopamine D2 receptor; Glx, glutamine/glutamate ratio; 5-HT_2A_, 5-hydroxy-tryptamine receptor type 2A.

Neuroimaging Techniques	Neurotransmitters Pattern	Diagnosis Groups	Brain Regions	Functional/Anatomical Alteration	Reference
^1^H-MRS	Glutamate	OCD	ACC	Decrease in N-acetylaspartate resonance peaks	[67]
		OCD	ACC and striatum	Decrease in glutamate peaks Increased in Glx peaks	[67] [57]
		Schizophrenia	ACC	Decrease in N-acetylaspartate resonance peaks	[75]
SPECT	Dopamine	OCD	Left caudate nucleus	Decrease in D2R binding	[83]
PET	Dopamine	OCD	Striatum	Decrease in [^11^C]Raclopride, a selective D2R antagonist, uptake	[73]
	Serotonine		Polar, dorsolateral, and medial frontal cortex	Decrease in [^11^C]MDL, a selective 5-HT_2A_ antagonist, uptake	
Resting state fMRI	-	OCD and schizophrenia	Hippocampus and the left posterior cingulate cortex	Abnormal local spontaneous neural activity	[84]
Trait-based spatial statistics and probabilistic tractography	-	Schizo-obsessive	Right sagittal layer and left crescent of the fornix/stria terminalis	Reduced fractional anisotropy and increased radial diffusivity resulted in altered connections in the default mode network, subcortical network, attention network, task control network, visual network, somatosensory network, and cerebellum	[85]
fMRI	-	Clozapine/olanzapine-induced OCS in schizophrenia	CSTC loop, left parahippocampal gyrus, globus pallidus, and the right precentral gyrus	Increased brain region activation	[89]

## Data Availability

All data are available upon request.
